# Relationship Between CNS Infections, Stroke, and Neurological Complications: A Case Series

**DOI:** 10.7759/cureus.78558

**Published:** 2025-02-05

**Authors:** Karan Soni, Erin S Fannin, Nojan Valadi

**Affiliations:** 1 Neurology, Philadelphia College of Osteopathic Medicine, Suwanee, USA; 2 Internal Medicine, East Alabama Medical Center, Opelika, USA; 3 Neurology, East Alabama Medical Center, Opelika, USA

**Keywords:** central nervous system infections, coccidioidal meningitis, hiv, neurological complications, neurosyphilis, stroke

## Abstract

Central nervous system infections and complications such as meningitis and stroke in immunocompromised patients can be caused by a wide spectrum of pathogens, including bacteria, viruses, parasites, or fungi. This case series first presents a case of a 24-year-old Latino male patient with HIV, cytomegalovirus (CMV) encephalitis, a positive CSF for *Coccidioides* antigen, and a stroke, who presented to the primary care office with a headache and double vision. With his symptoms now occurring for more than two months without improvement, the patient was sent to the ED for a repeat MRI, where an enlargement of his ventricles compatible with hydrocephalus was observed. This patient was diagnosed with coccidioidal meningitis and sent to a different facility for neurosurgical management. The second case describes a 28-year-old White male patient living with HIV and struggling with polysubstance use, who presented to the ED with a stroke and encephalopathy. Labs to address his underlying cause of encephalopathy showed a positive rapid plasma reagin (RPR) titer, and MRI results were consistent with bilateral acute ischemic infarcts of syphilitic vs. HIV-associated vasculitis. The patient was placed on aspirin, corticosteroids, and high-dose antibiotics. A prognosis of coccidioidal meningitis and neurosyphilis can be fatal if left untreated; as a result, this case series emphasizes the need for prompt stroke management and workup, especially in the context of infection and/or encephalopathy. Discovering the underlying cause of stroke can not only address the patient’s current symptoms but also prevent future stroke occurrence.

## Introduction

It is estimated that less than 15% of all strokes occur in patients aged 45 and younger. However, this number is on the rise, and the increase is frequently attributed to risk factors commonly seen in the older population such as hypertension, hyperlipidemia, diabetes, and obesity. There are several other factors including medication, alcohol, and drug use, as well as viral, fungal, and bacterial etiologies that should also be considered following presentation with stroke in this younger population. This series highlights the rising incidence of stroke in young adults due to various infections and related complications.

*Coccidioides* refers to a genus of fungi that are dimorphic in nature. Two species within this genus include *Coccidioides immitis* and *Coccidioides posadasii*. These fungi are endemic to the arid regions of the Western hemisphere and can lead to a fungal infection known as coccidioidomycosis. Symptoms can range from fever and cough to weight loss and a transient maculopapular rash. *Coccidioides* dissemination to the central nervous system (CNS) is common in immunocompromised patients, involving meningitis, stroke, and hydrocephalus as possible complications among others [1].

In comparison, *Treponema pallidum* is a spirochete that can cause the bacterial infection of syphilis. While the staging of this sexually transmitted disease can influence symptom presentation, spread to the CNS can occur at any time after infection. This term is referred to as neurosyphilis and can range from being asymptomatic and meningeal within the first year of infection to meningovascular in nature and then tabes dorsalis later on in disease progression. From a pathophysiology perspective, neurosyphilis involves the development of a lymphocyte and plasma cell-composed inflammatory infiltrate that can lead to nerve damage. Symptoms of neurosyphilis can vary but most often involve any combination of behavior changes, ataxia, stroke, headache, visual disturbances, hyporeflexia, or neuropathy [2].

In this case series, two separate cases of stroke in the setting of CNS infections presented to our facility in a month. The first case describes a meningitis patient with a history of stroke and coccidioidomycosis as indicated by leptomeningeal enhancement on MRI and CSF positive for *Coccidioides* antigen. The second case describes a stroke patient with a history of neurosyphilis as indicated by a reactive CSF Venereal Disease Research Laboratory (VDRL) test and MRI showing bilateral basal ganglia stroke.

## Case presentation

Case 1

A 24-year-old Latino male patient living with HIV, who had previously suffered from cytomegalovirus (CMV) encephalitis, and right basal ganglia infarct presented to the primary care office with a chief complaint of headache and double vision. When this patient was previously hospitalized, his CSF was also positive for CMV and *Coccidioides* antigen, which was determined to be the etiology of the stroke. The patient then had a follow-up outpatient MRI done, which showed significant leptomeningeal enhancement along with diffuse enhancement of the basal cisterns surrounding the brainstem. At this time, the patient reported no new symptoms and that he would arrive for his follow-up appointment in the clinic as scheduled in four months.

During his in-office appointment, the patient reported having constant, squeezing headaches, double vision, and decreased appetite from the past one to two months. No relief was achieved with acetaminophen use, and the patient reported staying awake at night due to the pain. However, he denied any nausea, vomiting, diarrhea as well as seizures, cough, or congestion. His neurological exam in the office was still pertinent for residual left-sided hemiparesis from his stroke, causing him to ambulate with a cane. Due to the patient’s past medical history and current symptoms in the context of his previous MRI findings, he was sent to the emergency room for further workup and imaging.

Upon the patient’s admission to the ED, a CBC, comprehensive metabolic panel (CMP), and HIV RNA level were ordered. Vital signs taken were within limits. Despite his chronic symptoms, the patient was in no acute distress. Pertinent positives on a general physical exam included bilaterally blurred disks without papilledema on the funduscopic exam, increased tone in the left hemibody, and tenderness in the left lower quadrant on palpation. Neurologic exam findings involved 5/5 strength in the right upper and lower extremities compared to 4/5 strength in the left upper and lower extremities. The patient also had increased tone in the left upper and lower extremities along with 3+ left biceps, triceps, and brachioradialis reflexes. He also had 2+ brisk patellar and Achilles reflexes on the left compared to 1+ patellar and Achilles reflexes on the right. The patient’s toes were upgoing on the left compared to downgoing on the right. He also had an asymmetrical gait with slow speed and step asymmetry.

A lumbar puncture was performed, producing about 15 mL of hazy CSF, which was collected across four tubes and then sent for studies. While the patient was still in the ED, results came back indicating CSF WBCs of 76 (elevated) that were lymphocyte predominant, three RBCs, 257 protein (elevated), and 30 glucose (decreased). CBC results were pertinent for an elevated neutrophil level of 84, while his HIV RNA levels also came back at <20 copies/mL, indicating the presence of HIV-1 but at a level lower than the lower limit of the assay.

**Figure 1:**
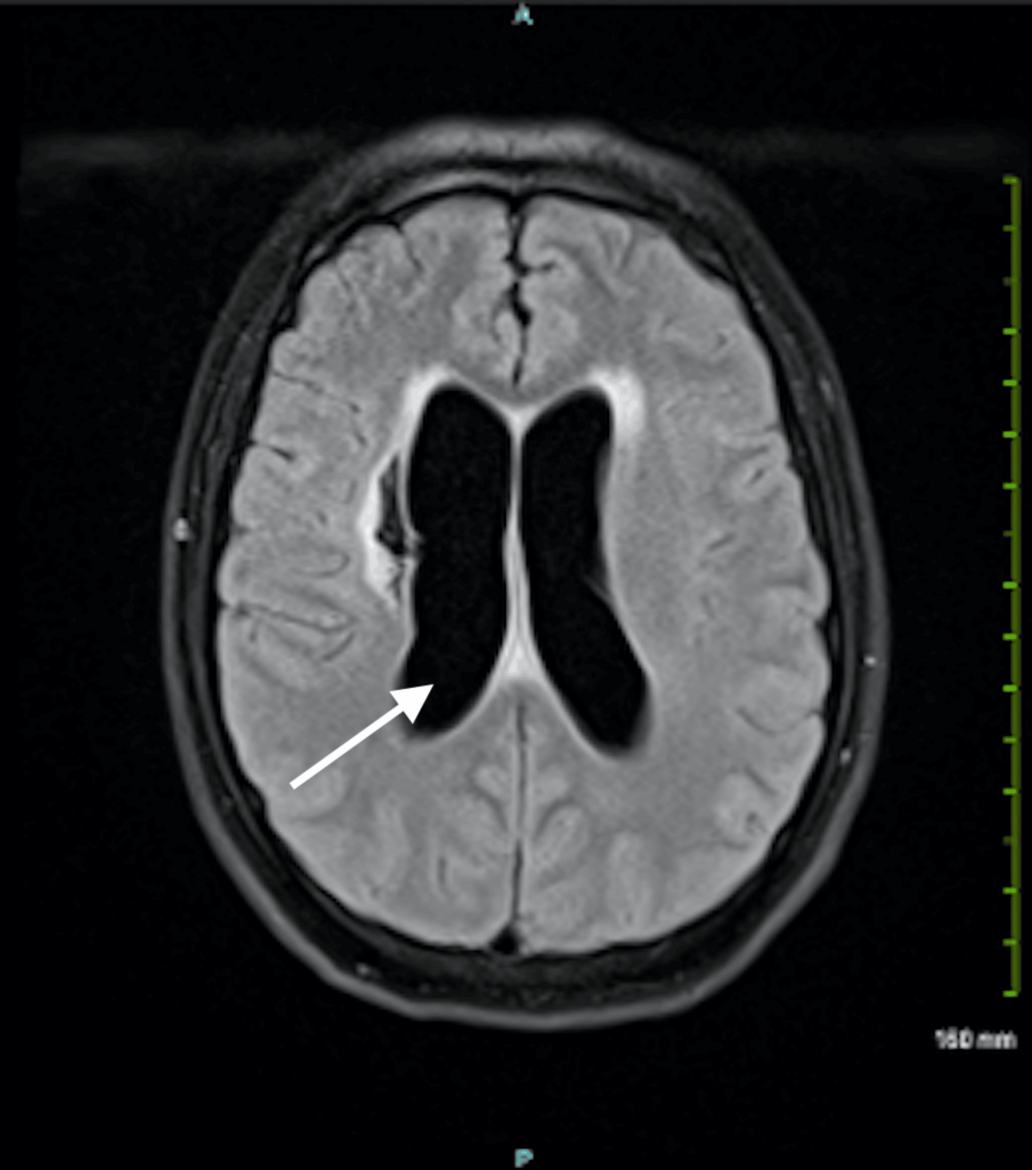
MRI of the head in a patient with coccidioidal meningitis, demonstrating significant ventriculomegaly (arrow) and a complication of hydrocephalus

A new MRI was compared to his results from four months ago. MRI of the brain demonstrated persistent enhancement of the basal cisterns and Sylvian fissures as well as leptomeningeal enhancement; however, his new MRI also demonstrated significant ventriculomegaly and hydrocephalus, which was not present four months ago (Figure 1).

A BioFire meningitis/encephalitis panel on the CSF was ordered to evaluate for an underlying cause of the patient’s meningitis. All pathogens came back negative, including a cryptococcal antigen on cryptococcal serology. At this point, bacterial and fungal cultures as well as a gram stain were ordered, and recommendations were given to start the patient on vancomycin and ceftriaxone. With the patient’s history of coccidioidomycosis, a diagnosis of coccidioidal meningitis was considered. An infectious disease consult was placed, and a transfer to a facility with neurosurgical services was initiated to address the patient’s ventriculomegaly and hydrocephalus, which was most likely due to an infectious process or possible subacute obstruction. In the meantime, the patient was to continue his low-dose aspirin, due to his stroke history, and his antiretroviral medications for HIV management.

Case 2

**Figure 2:**
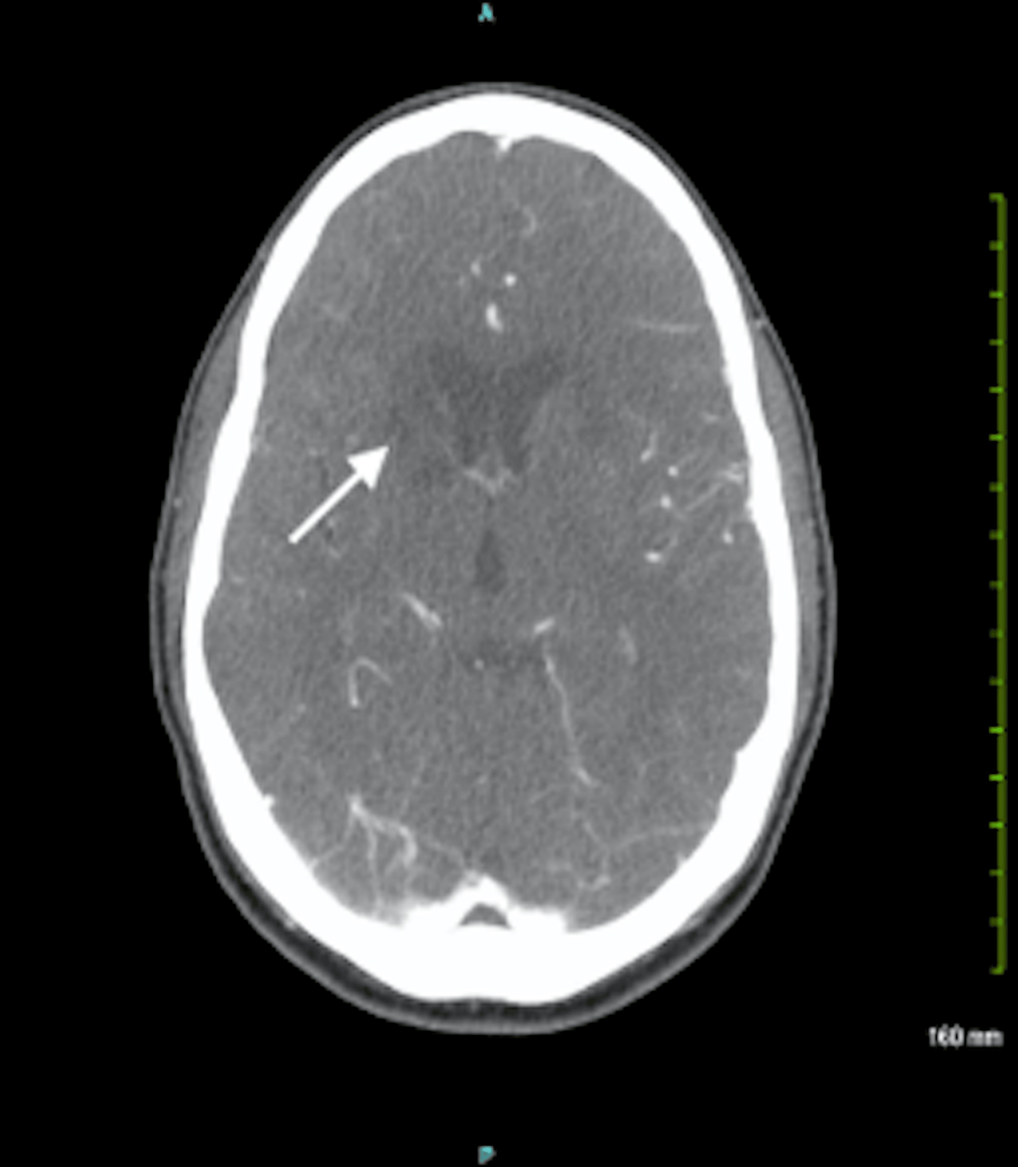
CT imaging of the head, demonstrating a right basal ganglia infarct (arrow), resulting in contralateral (left-sided) neurologic manifestations

A 28-year-old White male patient with a history of HIV and polysubstance abuse presented to the ED for stroke evaluation. The patient was found unresponsive the day before and was taken to a local medical center, where a CT of the head showed a basal ganglia infarct (Figure 2). He was transferred to the current site of this case presentation for further stroke management.

On presentation to the ED, the patient’s labs revealed mild leukocytosis. Given that the patient was encephalopathic on the exam, reactive oxygen species (ROS) was considered unreliable. On general physical exam, a 2/6 systolic ejection murmur was auscultated. Neurologic exam findings included sparse verbal output, limited left gaze with retraction nystagmus, diminished bulk and tone bilaterally, 5/5 strength in the right upper and lower extremities, and 4/5 strength on the left side, with notable asymmetry of strength on the left side. The patient had 2+ reflexes on his right side but 3+ reflexes on the left. His toes were upgoing on the left and right; however, it was more significant on the left side.

Multiple labs for treatable and reversible causes of encephalopathy were collected. Pertinent positives from these tests included a reactive rapid plasma reagin (RPR) titer, a positive drug screen for amphetamines and cannabinoids, and elevated blood urea nitrogen (BUN) and creatine phosphokinase (CPK) levels. While no evidence of bacteria was seen on urinalysis, urine chemistry tests showed elevated protein levels.

Several differentials were considered as a cause of the patient’s acute ischemic stroke and guided further management. An echocardiogram was ordered to assess for an embolic source of stroke, and a CT angiography (CTA) of the head and neck was ordered to evaluate for vasculitis directly caused by either HIV, syphilis, or an opportunistic infection. A lumbar puncture was also done to obtain CSF for analysis with the BioFire meningitis and encephalitis panel. Furthermore, the CSF was sent for a VDRL test due to the patient being RPR-positive. As the patient was HIV-positive, CD4 count and viral load were also checked. After completing the initial workup, an MRI was also ordered, showing vasculitis possibly due to an infectious etiology.

CTA of the head and neck confirmed the suspicion of vasculitis due to an infection, showing multifocal bilateral vascular stenosis. With the patient also having had a history of stroke, he was started on 81 mg of aspirin once a day. Lumbar puncture results indicated meningoencephalitis and were positive for human herpesvirus 6 (HHV6), with 470 lymphocytic predominant pleocytosis. The patient was started on high-dose penicillin as empirical therapy for neurosyphilis, as well as high-efficacy antiretroviral therapy (ART) for HIV.

## Discussion

The patient in Case 1 was promptly sent to the ED from the primary care office, given his immunocompromised status, current symptoms, and history of a positive *Coccidioides* antigen. Furthermore, leptomeningeal enhancement of the basal cistern on previous MRI findings is a significant finding, being observed in more than 90% of cases in previous studies [3]. After discovering ventriculomegaly and hydrocephalus on the patient’s new MRI, multiple differentials were created, including chemical meningitis, carcinomatous/lymphomatous meningitis, or other opportunistic infectious processes besides *Coccidioides*. As a result, a lumbar puncture was done to evaluate for infectious processes and to obtain CSF for the purpose of further analysis.

The differentials for the immunocompromised patient in Case 2 were much more extensive in the setting of encephalopathy and bilateral basal ganglia infarcts. These included HIV, syphilis, or opportunistic infection-associated vasculitis. Specific opportunistic infections included tuberculosis (TB), varicella zoster virus vasculitis, or even meningovascular syphilis, all of which could explain the patient’s MRI results [4]. However, given that the patient was RPR-positive, the number one diagnosis on the differential list was neurosyphilis. Furthermore, a negative workup using a BioFire meningitis/encephalitis panel along with no evidence of lymphoma, a clear chest x-ray, and predominantly normal findings on echocardiogram also supported this. A VDRL test on the CSF also eventually came back reactive as well.

An interesting finding during the workup of the patient in Case 2 was multifocal bilateral vascular stenosis on CTA, also known as the moyamoya phenomenon. This cerebrovascular condition involves occlusion of the terminal part of the internal carotid arteries and can be associated with acquired phenomena such as vasculitis. In the case of this patient, the 81 mg of aspirin he was put on for his history of strokes also serves as effective management for his vascular stenosis [5]. Another interesting finding was a positive HHV6 in the CSF. While neurosyphilis serves as the most likely underlying cause of the patient’s stroke, literature does support HHV6 possibly causing encephalitis. One study comparing HHV6 in immunocompromised to immunocompetent hosts found that while HHV6 in immunocompetent individuals was limited to infants younger than three years old, HHV6 can be reactivated in immunocompromised adults, leading to neurological complications such as encephalitis [6].

The main challenge encountered in Case 1 was with further management of the patient. Even though the patient was in no acute distress, the presence of hydrocephalus in the setting of untreated meningitis and an HIV-1 infection had a high risk of mortality. While shunt placement to address hydrocephalus can be delayed until inflammation decreases, our team favored neurosurgical consultation and facility transfer for surgical correction as consistent with current literature [7]. In comparison, while the encephalopathy for the patient in Case 2 was likely due to his bilateral basal ganglia infarcts, the presence of multiple confounding factors made stroke management and etiology discovery a challenge. These specific factors included his HIV status potentially resulting in comorbid CNS infectious processes, polysubstance abuse, a positive HHV6 in CSF, and a positive RPR titer. However, the MRI findings allowed for neurosyphilis to remain at the top of the differentials, leading to penicillin use as first-line management [8].

As a result, this case series supports much of the existing literature on the topic of how CNS infections of varying types can lead to neurological complications. Furthermore, this case series corroborated our beliefs that prompt diagnosis and management of encephalopathy or an infection of the nervous system can prevent an increase in mortality, especially in the setting of untreated coccidioidal meningitis and neurosyphilis leading to stroke.

## Conclusions

This series served as clinical examples of how CNS infections can manifest as strokes and even neurological emergencies warranting further management. There is a need for prompt management and workup, especially in the context of an infection or encephalopathy. This workup is especially important since both coccidioidomycosis and syphilis can affect other organ systems besides the nervous system. As a result, discovering the underlying cause of stroke or meningitis can not only address the patient’s current symptoms but also prevent future stroke occurrences. Based on our clinical experience, we recommend prompt infectious disease evaluation and management in an immunocompromised patient presenting with acute-onset symptoms to best prevent spread to the CNS. This can be done by a combination of a CBC, lumbar puncture, CSF analysis, gram stain, cultures, and imaging as appropriate. Furthermore, beginning both anti-infective agents in addition to antiplatelet therapy can achieve the goal of addressing the underlying cause of the patient’s symptoms while also preventing the occurrence of recurrent strokes.
